# Effect of Antioxidant Therapy on Oxidative Stress In Vivo 2021

**DOI:** 10.3390/antiox11030448

**Published:** 2022-02-24

**Authors:** Anna Maria Fratta Pasini, Luciano Cominacini

**Affiliations:** Department of Medicine, Section of Internal Medicine D, University of Verona (Italy), Policlinico G.B. Rossi, Piazzale L.A. Scuro 10, 37134 Verona, Italy; luciano.cominacini@univr.it

Oxidative stress (OS) is an imbalance between the formation of reactive oxygen and nitrogen species and antioxidant defenses. Both endogenous and exogenous agents induce OS, which causes oxidative damage to each of the major cellular biomolecules (i.e., carbohydrates, lipids, proteins and nucleic acids). OS may also alter many signaling pathways, thus promoting inflammation, inducing apoptosis, deregulating autophagy and impairing mitochondrial function and many other mechanisms [1,2]. Thus, not surprisingly, OS is one of the most important determinants of aging-related processes and a common denominator in the onset and development of chronic diseases such as cardiovascular and neurodegenerative diseases and cancer [3,4]. Therefore, strategies targeting OS have been the focus of several studies conducted in the last decades to explore possible health benefits of antioxidant supplementation/pharmacological therapy [1,4]. Finally, a better comprehension of the signal transduction through which oxidants act as second messengers is necessary in order to provide a rationale pharmacological approach. The rationale for exogenous antioxidant supplementation arises from the need to preserve health in the general population, hampering the occurrence of an ill health, its worsening and complications. Unfortunately, the controversial results of antioxidant supplementation in different diseases (especially in cardiovascular disease) and in aging weakened these assumptions [1,5,6,7,8,9]. However, there are many concerns and limitations that can help to explain clinical failures. The different results may depend on what extent OS plays a role in a certain disease and on the stage of the disease, since antioxidant therapy may fail when other factors become prevalent in the progression of the disease [1]. In addition, the differences in trial designs render comparing available data difficult. On the other hand, the possibility that the antioxidant supplements used may not be able to reach adequate concentrations in vivo must not be forgotten [1]. A further limitation is OS status assessment: in fact, although different OS biomarkers, belonging to both oxidant and anti-oxidant counterparts, have been identified in many pathological conditions and in aging, reliable OS biomarkers are still lacking [4]. Despite the conflicting results of clinical studies, as previously reiterated [10], there is still a need to continue the field of OS research and antioxidant supplementation in order to tailor medical management to individual patient characteristics. 

This Special Issue concerning “The Effects of Antioxidant Therapy on Oxidative Stress in Vivo 2021” contains four contributions, three research articles and one review.

In the first contribution, Sayed et al. [11] analyzed the role of NOD-like receptor family pyrin domain containing 3 (NLRP3) inflammasome in cardiac aging and age-related cardiac sarcopenia and researched whether melatonin supplementation may exert a beneficial effect to prevent these alterations in mice. Melatonin in recent years has gained much attention as a cytoprotective agent with anti-inflammatory and antioxidative properties [12,13]. The results of this elegant study show that in wild-type mice at an age of 12 months, there were initial signs of heart sarcopenia, a reduction in cardiomyocyte number with enhanced compensated cardiomyocyte hypertrophy and increased expression of beta-myosin heavy chain (β-MHC). This picture further worsened in cardiomyocytes of 24-month-old mice with excessive collagen deposition and increased expression of the proinflammatory cytokines interleukin (IL) IL-1, IL-6 and of tumor necrosis factor-alpha. At variance with these results, cardiomyocytes of NLRP3-knockout mice showed significant reductions in age-related changes compared with wild type mice. Interestingly, melatonin treatment maintained the normal cardiomyocyte structure, re-established cardiomyocyte number and reduced β-MHC expression of cardiac hypertrophy. In addition, melatonin recovered mitochondrial structure, lowered apoptosis and multivesicular bodies generation and downregulated the expression of β-MHC, IL-1 and IL-6. This study is a further evolution of previous reports in which the authors had analyzed the role NLRP3 inflammasome and melatonin in age-dependent sarcopenia and mitochondrial dysfunction [14,15]. Here, the authors found that NLRP3 plays a crucial role also in cardiac age-dependent sarcopenia, a situation characterized by reduced physical performance and declined cardiorespiratory fitness in older individuals. Very interestingly melatonin, by working on inflammation and on secondary OS, may be a further therapeutic approach for age-related cardiac sarcopenia. The authors went in depth on the morphological aspects of cardiac sarcopenia and proposed NLRP3 suppression and melatonin as new therapeutic approaches for age-related cardiac sarcopenia. 

In the next interesting contribution, Dai et al. [16] analyzed the protecting effect of baicalein on carbon tetrachloride (CCl4)-induced acute liver injury and the underlying molecular mechanisms in mice. Baicalein is a natural product from the root of *Scutellaria baicalens* and it possesses many beneficial pharmacological properties, including anti-inflammatory, antioxidative, anti-macrobiotic and immuno-regulatory activities [17]. The results indicate that baicalein in mice significantly reduced CCl4-induced elevations of alanine aminotransferase and aspartate aminotransferase and improved histopathology damage. Interestingly, baicalein also decreased malondialdehyde levels and augmented glutathione levels in liver tissues. Moreover, baicalein supplementation suppressed the nuclear factor kappa-B pathway, activated the antioxidant erythroid 2-related factor 2/heme oxygenase 1 pathway and significantly reduced CCl4-induced apoptosis, inflammation and ferroptosis in liver exposed to CCl4. Baicalein supplementation improves CCl4-induced liver injury in mice by enhancing antioxidant defenses and inhibiting OS, apoptosis, inflammation and ferroptosis. This study is an excellent example of how baicalein can counteract primary OS induced by CCl4. In particular, the authors carefully analyzed signaling pathways induced by baicalein which contributed to enhance antioxidant defenses. Since to date, safer, effective hepatoprotective drugs remain an unmet medical need, the present study calls attention to the possible clinical utility of Baicalein for patients affected by acute liver disease. 

In the third paper, Biernacki et al. [18] assessed the effect of topical application of cannabidiol (CBD), an antioxidant and anti-inflammatory phytocannabinoid found in *Cannabis sativa* L., on redox balance and on the metabolic disturbances in the blood of hairless rats chronically irradiated with UVA or UVB rays. In this context, previous evidence has shown that UV radiation (especially UVA) reaches microvessels, and it has the ability to directly affects blood vessels and internal tissue, particularly after chronic exposure [19]. Moreover, both UVA and UVB radiations increase reactive oxygen species (ROS) production in skin cells, which may spread to other tissues [20]. A first result of this sophisticated study is that CBD chronically applied to rat skin penetrated the bloodstream and is present in plasma and in membranes of polymorphonuclear leukocytes (PMNs). Furthermore, CBD reduced ROS generation and increased antioxidant defenses both in whole blood and in PMNs of rats subject to UV radiation. These effects of CBD were associated with a decrease in UV-induced lipid peroxidation, as well as protein modifications. The results suggest that CBD applied topically to the skin minimizes redox changes not only at the skin level but also at the systemic level. This is an interesting and complete study in which the authors not only measured CBD in plasma and in PMNs of rats exposed to UV radiation but also evaluated its effect on systemic ROS generation and antioxidant defenses. The results indicate that CBD may be useful in reducing systemic OS induced by UV radiation and suggest its potential for supporting therapies in other skin diseases. 

Finally, in their review, Fratta Pasini et al. [21] investigated whether ferroptosis may contribute to the process resulting in multiorgan damage, which often takes place during severe SARS-CoV-2 infection [22]. This hypothesis originated from the recent identification of ferroptosis signature in cardiac and renal tissues of a COVID-19 patient who died of cardiogenic shock [23]. Ferroptosis is a novel, iron-dependent nonapoptotic cell death that takes place via disproportionate peroxidation of polyunsaturated fatty acids (PUFAs) in cell membranes [24]. Growing evidence has demonstrated that ferroptosis plays significant pathogenetic roles in determining organ damage in many diseases and has become the focus of investigation to improve their prognosis and treatment [25]. In the first part of the review, the authors carefully analyzed the complex mechanisms of ferroptosis resulting in organ damage and provided evidence that altered iron metabolism, reductions in glutathione (GSH), decline of glutathione peroxidase 4 (GPX4) and increase in PUFA peroxidation by ROS are preeminent in the beginning and progression of ferroptosis [24,26]. Similarly, SARS-CoV-2 infection is characterized by intracellular iron trapping, inefficient GSH/GPX4 axis and increased ROS generation [26], all conditions that may boost hydroxyl radical formation via the Fenton reaction and PUFA peroxidation, which finally promotes ferroptosis. In the last part of the review, the authors examined potential therapeutic approaches by looking at every single mechanism of ferroptosis that can be counteracted by specific pharmacological interventions. In conclusion, given the increasing recognition of the key role of lipid peroxidation both in SARS-CoV-2 infection and in ferroptosis, the authors believe that there is a strong potential rationale for approaches directed at the prevention of lipid peroxidation in different clinical settings. 

We would like to express gratitude for the authors that contributed to this Special Issue. All studies underlined the pathophysiological role of OS and the potential benefits of antioxidant supplementation in different pathological circumstances. Likewise, all authors emphasized the requirement of further in vitro and in vivo studies to better explain the role of OS in various diseases and to propose effective and highly targeted therapies.
